# Papillary and peripapillary vascular densities and corresponding correlation with peripapillary retinal thicknesses using optical coherence tomography angiography in healthy children and adolescents

**DOI:** 10.1038/s41598-023-50934-3

**Published:** 2024-03-04

**Authors:** Fariba Ghassemi, Farhad Salari, Vahid Hatami, Masoumeh Mohebbi, Siamak Sabour

**Affiliations:** 1 Eye Research Center, Farabi Eye Hospital, Tehran University of Medical Sciences, Qazvin Square, Tehran, 1336616351 Iran; 2 Retina and Vitreous Service, Farabi Eye Hospital, Tehran University of Medical Sciences, Tehran, Iran; 3 Cornea Service, Farabi Eye Hospital, Tehran University of Medical Sciences, Tehran, Iran; 4 Safety Promotion and Injury Prevention Research Centre, Shahid Beheshti University of Medical Sciences, Tehran, Iran; 5 Department of Clinical Epidemiology, School of Public Health and Safety, Shahid Beheshti University of Medical Sciences, Tehran, Iran

**Keywords:** Retina, Retina

## Abstract

To evaluate the peripapillary retinal thickness (PPRT), vascular density (PPVD), and disc vascular density (PVD) and their correlations in normal healthy children using optical coherence tomography angiography (OCTA). This was a cross-sectional study of 70 eyes from 36 normal healthy children aged 6–18 years who performed optic nerve head scans using OCTA. The PPRT included the peripapillary nerve fiber layer (PP-RNFLT), inner retina (PP-IRT), middle retinal thickness, and outer retinal thicknesses. The PP-RNFLT and PP-IRT were not significantly different between males and females. Superior nasal peripapillary RNFLT and IRT were significantly affected by age (ANOVA, *P* > 0.05). The PP-IRT and PP-RNFLT were lower in the 7–11 years old group in comparison with the other 3 groups (Post hoc Tukey test, *P* value < 0.05). Age and sex-matched PVD were not correlated with PPVD (partial correlation, *P* > 0.05). PPRT was not correlated with PVD, PPVD, superficial and deep retinal vascular densities, and choroidal vascular density. This study demonstrated that PPRT appears to change during growth in childhood. Superior nasal PPRT was affected more in the groups, decreasing from less than 7 years old to 7–11 years old and then back to pre-reduction values after 11 years old.

## Introduction

Optic neuropathies can be associated with vascular pathologies and examining vascular perfusion is crucial in clinical practice^1^. The study of optic nerve head (ONH) perfusion has been recently popularized after progression in understanding glaucoma and ONH disease pathogeneses. It has been revealed that optic nerve circulation and anatomy may be altered by ocular diseases like high myopia^2^, infantile nystagmus^3^, optic disc drusen^4^, uveitis^5^, optic neuritis^6^, optic neuropathies^7^, glaucoma^8,9^, central nervus system (CNS) related disorders such as migraine^10^, epilepsy^11^, CNS tumors^12,13^, and systemic conditions like diabetes^14^ and thalassemia^15^ or drug induced changes^11,16^ in both children and adults^12,17,18^. Prompt diagnosis and management are crucial to prevent further visual impairment and promote optimal visual development. Pediatric ophthalmologists or neurologists typically oversee the evaluation and management of optic nerve circulation problems in children. Thus, a thorough understanding of optic nerve vascular anatomy and the availability of normative data is of paramount importance.

Optic nerve head perfusion is unique and complicated. Anterior ONH is perfused by two distinct branches from the ophthalmic artery. Short posterior ciliary arteries supply the deeper prelaminar tissues and branches from the central retinal artery perfuse superficial layers of the optic nerve. In addition, a recent study using indocyanine green angiography demonstrated peripapillary choroids play a role in the supply of prelaminar ONH^19^.

Optical coherence tomography angiography (OCTA) is an accurate noninvasive reproducible imaging technique developed based on OCT that qualitatively assesses the microcirculation within and around the optic disc. As a result, retino-choroidal vascular diseases, their pathogeneses, and new treatments can now be described and quantified. Healthy and glaucomatous eyes have been distinguished by the degree of vascular density (VD) using OCTA^19^. OCTA metrics are highly influenced by age, so we investigated the changes in papillary and peripapillary retinal microcirculation in healthy children and adolescents. Although many studies have evaluated ONH perfusion in the normal and diseased eye^20–23^, there is limited knowledge regarding the natural course of papillary and peripapillary vessel evolvement during development in childhood.

We aim to report a normative database on VD and layered retinal thickness (RT) of ONH area in normal healthy children and adolescents and to evaluate the correlation between peripapillary VD (PPVD) and papillary VD (PVD) and layered RT.

## Method

### Participants

This cross-sectional study was performed at Farabi Eye Hospital, a university hospital at Tehran University of Medical Sciences, from April 2018 to August 2021. This study followed the tenets outlined in the Declaration of Helsinki protocol and was approved by the Tehran University of Medical Sciences Review Board (IR.TUMS.VCR.REC.1397.1054). Healthy children and adolescents aged 7 to 18 years who were presented to the ophthalmology clinic for a routine examination, were enrolled in the study. Verbal informed consent from the participants and written consent from their parents or guardians was obtained prior to subjects’ participation in the study. Four groups of patients were defined: children less than seven, 7 to 10, 11 to 14, and more than 18 years old.

The inclusion criteria were all consecutive school-aged children and adolescents with best corrected visual acuity (BCVA) of 20/20, a spherical equivalent (SE) of − 0.5 to + 0.5, and an intraocular pressure of less than 21 mmHg. For the cases under 5 years old, cycloplegic refraction/spherical equivalent was checked. A normal appearing optic nerve head (ONH) and nerve fiber layer and symmetric ONH between left and right eyes were the necessity. Exclusion criteria included any condition that prevented accurate retinal imaging such as gesture problems and frequent blinking, systemic medical, surgical, and drug history, neurological, or ocular disease history, cataract, previous laser or ophthalmic surgery, and amblyopia.

### Imaging

**Figure 1:**
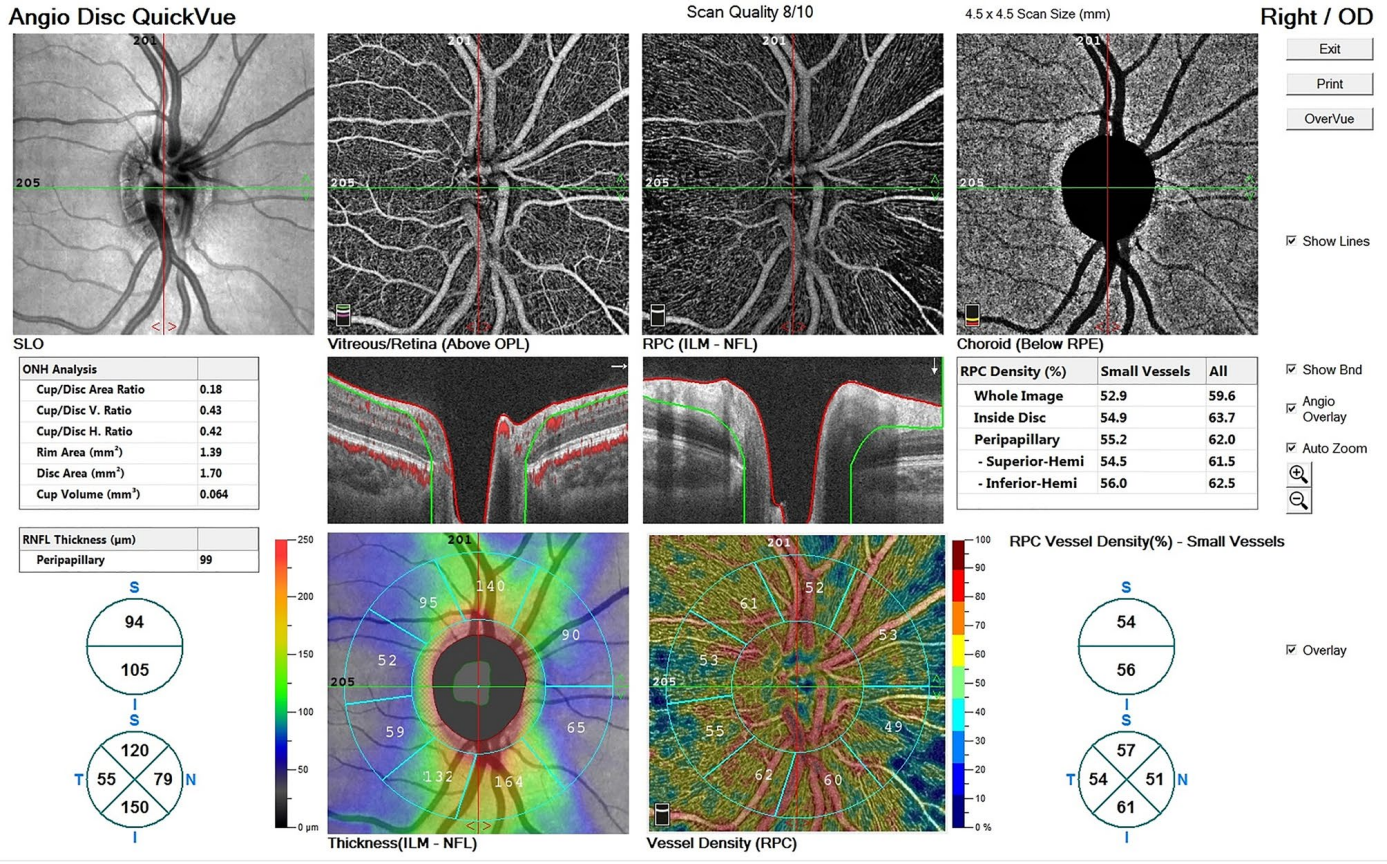
This figure shows a sample 4.5 × 4.5 mm images printout of optic nerve head vasculature and RPC vessel density [angiography (OCTA) device (RTVue XR Avanti, Optovue Inc., Fremont, California, USA)]. Papillary, peripapillary and zonal segmentation (temporal, superior, nasal and inferior) of Optic nerve head are also presented in this printout.

A single experienced operator took images using the AngioVue OCTA system version 2018,0,0,18 (Optovue RTVue XR Avanti, Optovue Inc., Freemont, California, USA). All Imaging was performed between 8 to 12 a.m. The light source of this device has a wavelength of 840 nm with a speed characteristic of 70,000 A-scans per second in the instrument. The scans consisted of a 4.5 × 4.5 mm disc map centered on the optic disc. It uses 304 vertical (Y-FAST) and horizontal (X-FAST) lines in the scanning area to make a 3D data cube and reduce the motion artifacts (Fig. 1). The volumetric scans were processed by the split-spectrum amplitude-decorrelation angiography algorithm (SSADA). The capillary density of ONH was calculated as a percentage of blood vessels in the specified region and the inbuilt software divided it into the inside disc (papillary) region (PVD) and peripapillary region (PPVD). The device automatically determines the retinal peripapillary capillary plexus (RPC) slab, from ILM to the outer limit of RNFL, Superior capillary plexus (SVD) at 3 μm below the internal limiting membrane (ILM) of the retina, and the outer boundary at 15 μm beneath the inner plexiform layer (IPL), the Deep capillary plexus(DVD) at 15 μm beneath the IPL to 71 *μ*m under the IPL and choroidal capillary plexus(CVD) was determined as the area between 15 to 45 μm below the Bruch’s membrane.

The disc margin for each subject was automatically delineated along the neural canal opening using the OCT images, and if any artifact occurred manual correction was performed. A 1.5 mm diameter circle was centered on the disc to evaluate PVD and a larger concentric circle with a diameter of 3.4 mm was used to measure PPVD. We report and analyze the PVD and PPVD data after the subtraction of large vessels by inbuilt software. Poor-quality scans (scans with signal strength index below 55) or registered image sets with residual motion artifacts or other artifacts (discontinuous vessel pattern) were excluded from the analysis.

The software calculates the peripapillary thickness of RNFL (PP-RNFLT) between the internal limiting membrane (ILM) and the outer boundary of the nerve fiber layer (NFL), peripapillary inner retinal thickness (PP-IRT) between ILM and the outer boundary of the inner plexiform layer (IPL), peripapillary middle retinal thickness (PP-MRT) from the outer boundary of IPL to the outer boundary of the outer plexiform layer (OPL). The peripapillary outer retinal thickness (PP-ORT) was calculated from the outer boundary of OPL to the outer boundary of the retinal pigment epithelium (RPE- by subtracting this from the ILM to RPE thicknesses).

Moreover, we calculated the global value and the inbuilt 8 segments PP-RNFLT values as nasal-superior (NS), nasal-inferior (NI), inferior-nasal (IN), inferior-temporal (IT), temporal-inferior (TI), temporal-superior (TS), superior-temporal (ST) and superior-nasal (SN) according to the instrument software (Fig. 1). For the analysis, we used OCT scans acquired at the first visit.

### Statistical analysis

Statistical analyses were performed by statistical software (SPSS software Version 21; SPSS, Inc., Chicago, IL, USA). All normally distributed data was reported as the mean with standard deviation and non-normally distributed data was presented as the median with an interquartile range. Kolmogorov–Smirnov test and histogram were used to access the distribution. General estimating equations (GEE) was utilized to account for correlation between two eyes of each subject. A partial correlation coefficient controlling for age, sex, and BMI were used to evaluate the univariate linear correlation between VD and RT. The required sample size for a 5% significance level and 90% power regarding a correlation coefficient of 0.62 between RNFL thickness and peripapillary flow density was 25(Alnawaiseh28). We included data from 70 eyes from 36 normal healthy children in the analysis. A *P* value less than 0.05 after adjustment with Sidak technique was considered statistically significant.

### Ethics approval and consent to participate

This study was conducted under general approval from the Institutional Review Board of Tehran University of Medical Sciences.

## Results

### Demographic data

This study enrolled 70 eyes of 36 subjects, including 27 boys (75%) and 9 girls (25%). The mean disc and cup areas were 1.9 ± 0.3 mm^2^ and 0.17 ± 0.25 mm^2^, respectively. The mean age was 10.86 ± 3.46 years, with a range of 6 to 18 years.

### Peripapillary retinal thicknesses

**Figure 2:**
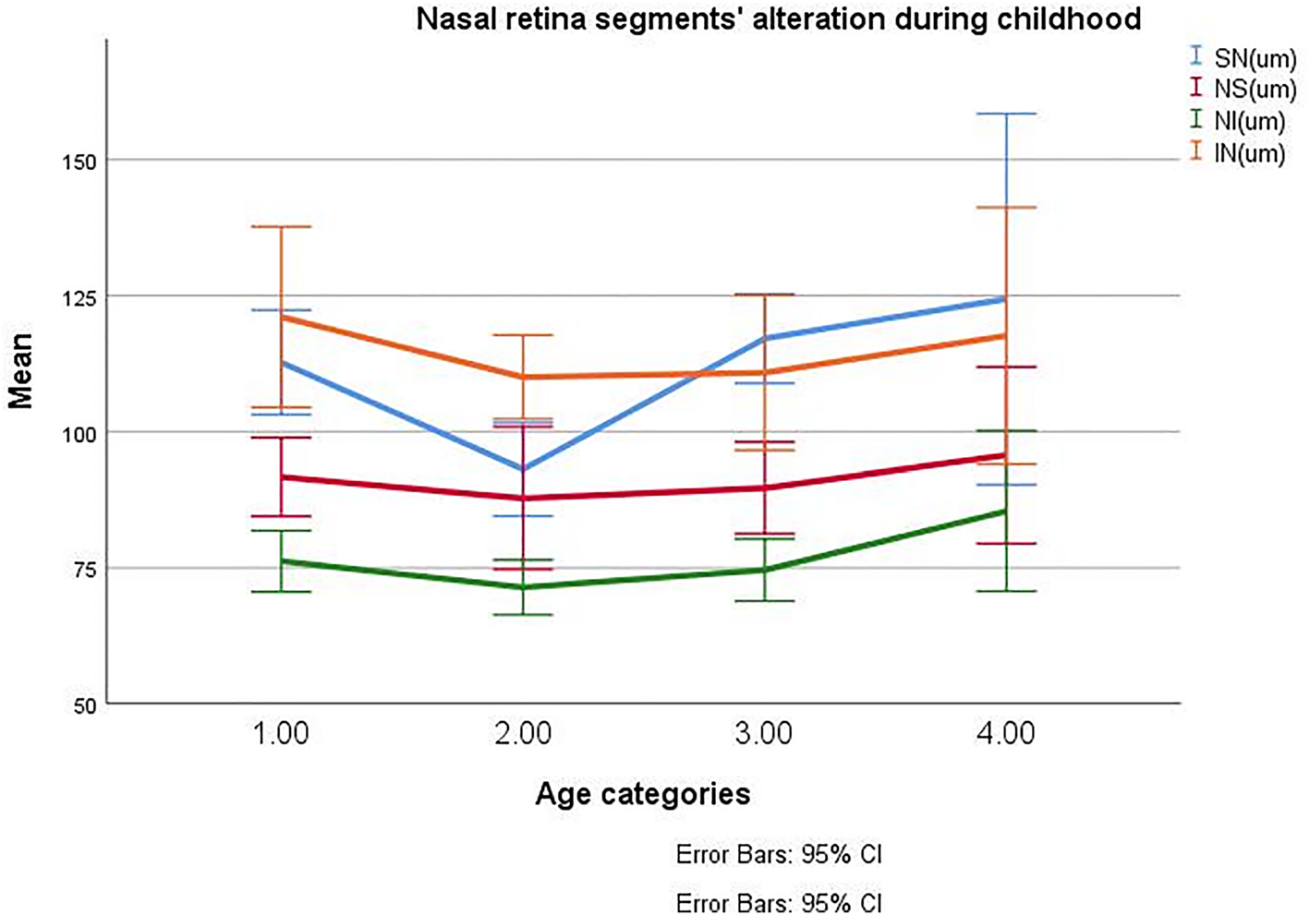
Nasal peripapillary segments retinal thickness alteration during childhood and adolescence.

The peripapillary retinal thickness (PPRT) in different parts has been summarized in Table 1, Fig. 2. The PP-RNFLT followed the ISNT rule in these children and adolescents and was the highest in the inferior sector and the lowest in the temporal sector. Other sectoral retinal layer thicknesses did not pursue this rule. In terms of PPRT, the corresponding mean whole PP-RNFLT, PP-IRT, PP-MRT, and PP-ORT were 99.87 ± 10.41, 145.2 ± 12.9, 48.0 ± 3.35, and 111.8 ± 6.61) μm. The PPRT was similar in both eyes (*P* > 0.05). The PP-RNFLT, PP-IRT, PP-MRT, and PP-ORT were not significantly different between males and females (GEE, all *P* > 0.05).

**Table 1:** Peripapillary retinal thickness of inner, mid, and outer retinal thicknesses in healthy children and adolescents categorized into 4 age groups.

Thickness (um)	Age categories of the study population				*P* value
	Younger than 7 years (N = 8)	7–11years (N = 16)	11–14 year (N = 20)	Older than 14 years (N = 8)	
PP-RNFL					
Whole	101.06 ± 12.1	97.86 ± 8.2	100.56 ± 9.48	101.2 ± 14.62	0.883
TS	76.22 ± 11.03	79.87 ± 9.73	76.27 ± 9.83	70.72 ± 6.52	0.064
ST	129.58 ± 19.96	130.31 ± 14.46	133.85 ± 13.24	124.79 ± 14.85	0.498
SN	113.5 ± 17.09	93.07 ± 19.24	122.23 ± 20.46	124.39 ± 38.14	0.002
NS	90.7 (81.5, 102.8)	86.1 (78.275, 90.7)	90.5 (74.05, 99.475)	94.8 (87.05, 103.15)	0.727
NI	75.54 ± 10.19	71.4 ± 11.41	74.02 ± 10.46	84.7 ± 16.6	0.438
IN	121.05 ± 30.01	110 ± 17.41	110.82 ± 27.76	117.6 ± 28.25	0.774
IT	136.71 ± 24.73	141.79 ± 15.19	135.76 ± 17.73	131.53 ± 31.53	0.571
TI	62.2 ± 9.99	64.62 ± 10.35	65.96 ± 10.43	62.37 ± 6.3	0.594
PP-IRT					
Whole	146.08 ± 14.58	144.45 ± 9.68	144.8 ± 12.16	146.71 ± 19.3	0.984
TS	126.54 ± 14.18	131.03 ± 10.34	126.2 ± 12.7	121.56 ± 9.23	0.236
ST	174.13 ± 24.73	176.7 ± 16.7	178.53 ± 15.91	170.13 ± 18.01	0.703
SN	157.24 ± 18.27	134.98 ± 20.52	163.61 ± 22.4	166.8 ± 39.49	0.003
NS	138.1 (121.1, 144.275)	128.55 (121.55, 133.025)	131.4 (113.925, 140.575)	137.9 (125, 149.4)	0.688
NI	116.99 ± 10.6	115.14 ± 12.41	114.16 ± 11.36	126.31 ± 19.02	0.568
IN	161.17 ± 30.71	152.33 ± 18.12	150.59 ± 30.67	158.86 ± 33.05	0.818
IT	176.5 (168.4, 205.8)	185.85 (175.55, 207.925)	179.2 (165.65, 191.4)	188.7 (178.45, 192.5)	0.304
TI	117.53 ± 12.82	122.54 ± 11.97	121.12 ± 12.11	118.61 ± 12.41	0.759
PP-MRT					
Whole	47.5 ± 5.01	48.74 ± 3.06	47.57 ± 2.16	48.07 ± 2.76	0.54
TS	52.35 (48.35, 59.325)	53.4 (52.4, 55.6)	54.2 (52.35, 57.25)	59 (49.6, 60.2)	0.871
ST	46.86 ± 6.47	47.85 ± 5.01	46.24 ± 3.02	46.93 ± 4.01	0.611
SN	45.02 ± 6.15	45.85 ± 4.17	43.83 ± 2.26	44.34 ± 4.76	0.263
NS	43.4 (38.475, 45.75)	43.2 (40.7, 45.75)	43.7 (40.425, 45.275)	41 (40.2, 43.35)	0.25
NI	43.87 ± 4.56	44.43 ± 3.64	43.06 ± 3	42.32 ± 1.67	0.192
IN	43.64 ± 4.18	43.87 ± 3.61	42.88 ± 3.46	45.81 ± 4.22	0.45
IT	48.7 (43.3, 52.1)	45.3 (44.125, 51.175)	45.1 (42.9, 48.3)	47.25 (44.25, 49.85)	0.495
TI	60.1 (55.3, 65.45)	58.9 (58.3, 63.7)	58.9 (57.65, 60.3)	61 (50.75, 67.35)	0.67
PP-ORT					
Whole	108.78 ± 8.3	113.48 ± 4.54	111.97 ± 7.04	112.74 ± 5.75	0.48
TS	111.09 ± 9.24	114.05 ± 7.66	111.93 ± 5.59	113.26 ± 6.88	0.74
ST	110.63 ± 8.61	114.71 ± 6.18	111.42 ± 6.32	112.07 ± 5.64	0.35
SN	112.82 ± 9.7	117.63 ± 7.56	114.5 ± 7.53	113.74 ± 6.64	0.44
NS	108.97 ± 10.85	116.6 ± 5.79	116.14 ± 8.4	113.69 ± 8.56	0.143
NI	110.54 ± 8.71	115.4 ± 8.36	115.36 ± 8.48	115.02 ± 5.59	0.509
IN	105.49 ± 9.1	110.95 ± 5.22	108.61 ± 8.69	111.58 ± 5.82	0.304
IT	102.55 ± 9.44	108.1 ± 4.86	105.46 ± 8.39	108.34 ± 7.36	0.293
TI	109.85 (99.75, 113.25)	108.6 (107.8, 112)	106.8 (104.2, 114.075)	110.2 (107.55, 119.45)	0.49

*PVD* papillary vascular density; *PPVD* peripapillary vascular density; *WiVD* whole image vascular density; *NS* nasal superior; *NI* nasal inferior; *IN* inferior nasal; *IT* inferior temporal; *TI* temporal inferior; *TS* temporal superior; *ST* superior temporal; *SN* superior nasal; *PP-RNFLT* peripapillary retinal nerve fiber layer; *PP-IRT* peripapillary inner retinal thickness; *PP-MRT* peripapillary middle retinal thickness; *PP-ORT* peripapillary outer retinal thickness.

*Variables with normal distribution were presented with mean ± standard deviation and non-normally distributed data were presented with median and interquartile range (Q1; Q3).

Symbol * shows a *P* value less than 0.05.

PP-RNFLT at SN showed a significant difference among these 4 groups (*P* < 0.001, GEE); the amount was lower in 7–11 years old group related to the other 3 groups (GEE with Sidak correction showed significant differences between groups (group 2 vs. 1, *P* = 0.04; group 2 vs. 3, *P* < 0.001).

The PP IRT was only significantly different between the 4 age groups at the SN segment (*P* < 0.001, GEE). Pairwise analysis with Sidak correction revealed that there was a significantly lower amount in 7–11 years old group related to the other 3 groups (group 2 vs. 1, *P* = 0.029; group 2 vs. 3, *P* = 0.004) (Fig. 2). However, the PP-MRT and PP-ORT did not significantly differ between the 4 categorized age groups.

### Papillary and peripapillary retinal vascular densities

**Figure 3:**
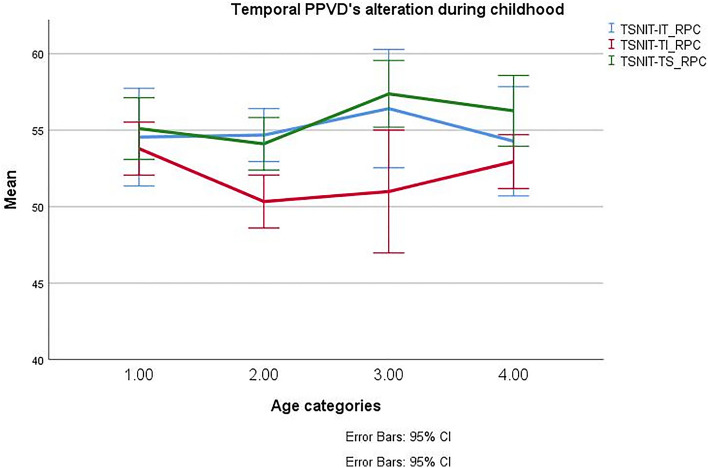
Temporal peripapillary segments radial retinal vascular density alteration during childhood and adolescence.

The PVD and PPVD in different parts of the ONH area have been summarized in Table 2. The PPVD evaluation showed a decreasing order at TS, IT, ST, TI, IN, NS, SN, and NI. The PVD and PPVD were similar in both eyes (GEE, *P* > 0.05). The PVD and PPVD were not significantly different between males and females (GEE, *P* > 0.05). Analysis didn’t show significant differences among the four categorized age groups except for the TI region of PPVD (*P* = 0.03, GEE). PPVD at TI was decreased from less than 7 years old to 7–11 years old (pairwise GEE with a Sidak correction, *P* value = 0.01) (Fig. 3). PVD was not correlated with PPVD after controlling for age and sex (*P* = 0.18, partial correlation).

**Table 2:** Papillary and peripapillary retinal vascular densities in healthy children and adolescents in 4 age groups.

Vascular Density (VD-%)	Age categories of the study population					*P* value
	Younger than 7 years (N = 8)	7–11 years (N = 16)	11–14 years (N = 20)	Older than 14 years (N = 8)	Whole patients mean	
WiVD	50.33 ± 1.82	49.15 ± 2.35	49.56 ± 1.22	49.97 ± 1.27	49.63 ± 1.98	0.586
PVD	51.94 ± 2.95	52.16 ± 3.67	50.87 ± 4.01	49.26 ± 3.28	51.51 ± 3.53	0.408
PPVD						
Whole	51.1 (49.9–52.5)	50.7 (48.4–52)	51.2 (50.6–52.19)	51.2 (49.8–52.7)	51.1 (49.8–52.2)	0.8
NS	49.31 ± 3.92	48.17 ± 3.4	48.26 ± 5.22	48.58 ± 2.49	48.54 ± 3.6	0.81
NI	48.55 ± 3.36	46.71 ± 4.74	46.84 ± 2.81	46.76 ± 3.03	47.2 ± 3.96	0.43
IN	50.21 ± 5.05	48.76 ± 5.22	49.16 ± 2.15	51.9 ± 3.61	49.65 ± 4.69	0.36
IT	53.2 (51.5–58.6)	53.42 (51.8–56.6)	56.1 (53.9–59.5)	53.1 (51.2–55.5)	53.8 (51.9–56.9)	0.56
TI	53.79 ± 2.73*	50.33 ± 3.89*	50.99 ± 3.83	52.94 ± 1.9	51.69 ± 3.62	0.010 (*)
TS	55.1 ± 3.17	54.11 ± 3.87	57.37 ± 2.08	56.26 ± 2.5	55.1 ± 3.45	0.051
ST	52.02 ± 4.24	53.55 ± 3.37	53.47 ± 2.85	51.16 ± 3.41	52.79 ± 3.58	0.39
SN	47.62 ± 3.63	49.35 ± 3.47	48.48 ± 3.23	48.09 ± 2.68	48.61 ± 3.36	0.67

Variables with normal distribution were presented with mean ± standard deviation and non-normally distributed data were presented with median and interquartile range (Q1; Q3). Symbol * shows a *P* value less than 0.05.

*PVD* papillary vascular density; *PPVD* peripapillary vascular density; *WiVD* whole image vascular density; *NS* nasal superior; *NI* nasal inferior; *IN* inferior nasal; *IT* inferior temporal; *TI* temporal inferior; *TS* temporal superior; *ST* superior temporal; *SN* superior nasal; *PP-RNFLT* peripapillary retinal nerve fiber layer; *PP-IRT* peripapillary inner retinal thickness; *PP-MRT* peripapillary middle retinal thickness; *PP-ORT* peripapillary outer retinal thickness.

### Papillary and peripapillary retinal vascular densities correlation with retinal thicknesses

Partial correlation controlling for age and sex was used to evaluate the correlation between peripapillary retinal thickness and retinal vasculature. PPRT was not significantly correlated with PVD nor was PPVD (Table 3). Also, PPRT is not significantly correlated with SVD, DVD, or CVD. However, there was a marginally insignificant positive correlation between PPORT and CVD (r = 0.38, *P* = 0.02, *P* values were corrected with the Bonferroni method, and a value less than 0.01 was considered statistically significant).

**Table 3:** The partial correlation coefficients of papillary and peripapillary vascular densities and thicknesses for different variables in healthy children and adolescents controlling for age and sex.

	PP-RNFLT	PP-IRT	PP-MRT	PP-ORT
PVD	− 0.048	− 0.026	0.127	− 0.094
*P* value	0.786	0.88	0.466	0.593
PPVD	0.087	0.166	0.285	0.154
*P* value	0.621	0.342	0.097	0.377
Whole image SVD	− 0.239	− 0.177	− 0.001	0.229
*P* value	0.167	0.31	0.996	0.186
Whole image DVD	− 0.292	− 0.202	0.066	0.352
*P* value	0.088	0.245	0.705	0.038
Whole image CVD	0.148	0.182	0.03	0.389
*P* value	0.397	0.295	0.865	0.021

*P* values were corrected with the Bonferroni method and a value less than 0.01 was considered as statistically significant.

*PVD* papillary vascular density; *PPVD* peripapillary vascular density; *SVD* superior vascular density; *DVD* deep vascular density; *CVD* choroidal vascular density; whole image vascular density; *PP-RNFLT* peripapillary retinal nerve fiber layer; *PP-IRT* peripapillary inner retinal thickness; *PP-MRT* peripapillary middle retinal thickness; *PP-ORT* peripapillary outer retinal thickness.

## Discussion

This study is the first study that evaluated the correlation between different layers of PPRT and PPVD in children and adolescents. In normal children and adolescents, papillary and peripapillary VD(s) and retinal vasculature were not correlated. Age was observed to have an increased influence on SN and NI RNFL thickness, SN of IRT as well as TI retinal vascular density within the age groups.

Vascular dysfunction is known to be a key factor in the pathogenesis of many ocular disorders such as diabetic retinopathy, glaucoma, ischemic and nonischemic optic neuropathies, and other ONH diseases. OCTA showed a probable cause-effect correlation between PPVD and debilitating glaucoma disease^24^. Although it is necessary to know the natural course of the cicumpapillary vascular system to efficiently utilize these data in clinical practice, there are few efforts to report data on optic nerve head OCTA in healthy children^25–27^. In the current study, we reported the measurements of PVD and PPVD, and PPRT using OCTA in normal healthy children and adolescents and their correlation. As a result of these data, we can assume that the development of peripapillary vessels and thickness is a continuous and fluent process during childhood.

As we expected the PP-RNFLT followed the ISNT rule in these children and adolescents and was the highest in the inferior sector and the lowest in the temporal sector. Other sectoral retinal layer thicknesses did not follow this rule. Similar to our study, a study by Liu et al.^28^ on normal adults showed that PPVD did not follow the “ISNT” rule. The highest PPVD was found in the temporal sectors where the RNFL was the thinnest. We disclosed that the PPVD showed a decreasing order at TS, IT, ST, TI, IN, NS, SN, and NI. The temporal region may be the site of the main blood supply and drainage so that the macula can meet its high metabolic requirements, leading to higher VD. Additionally, as a diffractive layer, the thinner the RNFL layer is, the more VD would be apparent in OCTA.

Analysis of data showed that the median whole PPVD and mean of PVD were 51.1 ± 2.7 and 51.51 ± 3.53 percent in our series. In a study by Icel et al.^1^ on healthy children, the mean PPVD was 52.5 ± 3.4%. Zhang et al.^22^ reported a mean PPVD of 51.66 ± 5.51% and a PVD of 51.72 ± 2.90% in 71 eyes of normal children. The similarity among measured values signifies probably racial variations may not affect peripapillary vascular density. In consensus with Icel et al. the PVD and PPVD were not significantly different between males and females. PVD and PPVD were unassociated after controlling for age and sex, a finding that has previously been observed in children^1,25^.

Furthermore, we classified children and adolescents into four age groups to examine possible changes in RT and VD during different ages in these groups, since previous studies on the macular structure had shown such significant changes^26^. From the first group to the second group, IRT decreased at the nasal retina. This decrease reaches statistical significance at the superior nasal segment (Table 1). The same finding in SN and NI was observed at PP-RNFLT. From less than 7 years to 7–11 years old, RNFLT decreased at the nasal retina. This decrease reaches statistical significance in SN and NI segments (Table 1). From 7–11 to 11–14, PP-RNFLT at SN increases significantly and reaches pre-reduction values, and then remains stable afterward (Fig. 2). No significant changes occurred in the PP-MRT or PP-ORT during these age courses. Analysis of data showed that PP-ORT at nasal subsegments (SN, NS, NI, and IN) increased in children from less than 7 years to the second group (7–11 years); however, this increase didn’t reach statistical significance.

On the other hand, we noticed similar changes in PPVD at temporal zones with a significant decrease of VD at TI from less than 7 years old to 7–11 years old (Table 2).

Although PVD and PPVD were not correlated with age in this study, four age groups showed the peripapillary vessel and RT change and more probable remodeling, is a continuous process during childhood that should be contemplated in the comparative studies. Previous research on adults showed some vascular changes occur from the third to the fourth decade^23,27^.

Regarding the effect of gender, the PP-RNFLT, PP-IRT, PP-MRT, and PP-ORT were not significantly different between males and females. Males and females have different RNFL anatomic characteristics, suggesting that physiologic differences may exist between them in terms of glaucoma prevalence^29,30^. A study by Wang et al.^31^, Li et al.^30^, Khawaja et al.^32^, and Wang et al.^33^ showed that male sex was significantly associated with decreased RNFL thickness in children^31^ and adults^30,32,33^, whereas other studies have found no significant association between RNFL thickness and sex^34–36^. Li et al. reported that females exhibited significantly thicker RNFLs than males at more than 40% of retinal locations, mainly in the T, ST, N, IN, and IT. Variability in these results may be attributed to different sample sizes and measurement techniques. As far as we are aware, this is the first study that evaluates MRT and ORT in children and adolescents; no comparable studies have been found.

The PVD and PPVD were not significantly different between males and females. Kiziltoprak et al.^37^ reported that IT and ST radial peripapillary capillary VD were significantly higher in girls than boys. The observed data on adults in our group did not show similar results in regression analysis^23^.

In this study, we evaluated the correlation between different layers of PPRT, and radial PPVD for the first time. PVD and PPVD did not show any significant correlation with PP-ORT. Due to the anatomical perspective of retinal perfusion, this irrelevance was expectable. The peripapillary capillary plexus is placed superficially in PP-RNFL and therefore its branches do not drench the outer retina^38^. Peripapillary ORT is likely to be more relevant to the choriocapillaris system, for it supplements. Our data showed a positive marginally insignificant correlation between CVD and peripapillary ORT. There was no significant correlation between PP-RNFLT and PPVD nor PVD, despite some articles in the literature showing that PPVD and RNFLT are strongly correlated^39–42^. Several discrepancies also exist between these two conclusions^28,42,43^. The reason behind this inconsistency could be explained by the different study populations, different definitions, and quantitation methods, different measurement instruments and imaging techniques, and different statistical analyses. Some studies have not differentiated the WI-VD (containing a rectangular area with a 4.5 mm side of the square having a circular area by 4.5 mm diameter surrounding the disc center, PVD (the circular region centered on the ONH), and PPVD (a circle surrounding PVD but not including it)^41^. Another plausible explanation for this discrepancy could be the role of continuing developmental process.

Similar to Liu et al.^28^ we showed that PVD was not significantly correlated with PPVD after excluding large vessels. This may be explained by the differences in capillary sources within the optic nerve head and the peripapillary area^28^. Although both the papillary and peripapillary areas are perfused by branches of the central retinal artery, studies have demonstrated vascular configuration significantly alters at various distances from the ONH^44^. Moreover, a recent study revealed that a centripetal flow from the peripapillary choroid is responsible for ONH tissue perfusion^19^.

These findings could improve our understanding of ONH microvasculature. In a study, Fernández-Vigo et al. evaluated the correlation between different peripapillary vascular layers [superficial capillary plexus (SCP), deep capillary plexus (DCP), and choriocapillaris (CC)] in healthy adults. They found that depending on the sector these layers could negatively or positively correlate to each other or even show no correlation. They found that in inferior sectors SCP significantly correlates to DCP while DCP negatively correlates to CC^27^. It seems that the variation in the correlation between different segments could be explicated by the difference in the contribution of choriocapillaris perfusion in these two segments.

OCTA studies have several limitations by their nature including automatic measurement of the values of VD and RT. This study was a cross-sectional study designed with small sample size. Also, we didn’t analyze sensitivity parameters. A projection-resolved algorithm used to suppress the projection artifact was not utilized in this study^22^. In order to clarify the influence of peripapillary VD on RT and the effect of aging on these values, well-designed longitudinal studies are needed.

In conclusion, according to this study, peripapillary retinal thickness appears to change segmentally during growth in childhood. This study revealed that the nasal peripapillary retinal thickness of children decreases from less than 7 years old to 7–11 years old and then back to pre-reduction values after 11 years old. Similar changes were noticeable in temporal peripapillary vascular thickness. Peripapillary retinal thickness was not significantly correlated with peripapillary vascular density and papillary vascular density.

## Data Availability

The data that support the findings of this study are available from the corresponding author (Fariba Ghassemi) upon reasonable request and after permission from Farabi Eye Hospital’s managing group and research center.
